# Feasibility of Virtual Blood Pressure Monitoring Titrated to ACC/AHA Thresholds Among Outpatients With Postpartum Hypertension

**DOI:** 10.1016/j.jacadv.2023.100390

**Published:** 2023-06-21

**Authors:** Amy A. Sarma, Daniela R. Crousillat, Paola Del Cueto, Nandita S. Scott, Jessica S. Tangren, Allison Bryant Mantha, Ilona T. Goldfarb

Hypertensive disorders of pregnancy (HDP) are a leading cause of postpartum readmission, morbidity, and mortality.^1^ There is significant interest in blood pressure (BP) home monitoring for outpatient postpartum hypertension management, but optimal frequency, duration, and BP targets have not been established. In this pilot study, we aimed to investigate the feasibility of postpartum BP titration to a target systolic BP (SBP) <130 mm Hg based on the American College of Cardiology (ACC) and American Heart Association (AHA) recommendations for nonpregnant patients.^2^

Sixty-two postpartum English and Spanish-speaking patients who delivered at Massachusetts General Hospital between February and May of 2021 with a diagnosis of chronic hypertension (cHTN), gestational hypertension (gHTN), or preeclampsia according to the American College of Obstetricians and Gynecologists (ACOG) criteria were recruited at labor hospitalization. Participants who did not own BP cuffs were provided with an automatic cuff and were educated on BP self-measurement at the time of study enrollment. Patients were also provided educational videos on how to measure BP in addition to education on hypertension and HDP that could be accessed at any time. After discharge, they reported self-measured BPs and received daily feedback, including medication initiation/titration according to a study algorithm that targeted a BP <130/80 mm Hg by 6 weeks postpartum. If diastolic BP remained >80 mm Hg after SBP control, patients were monitored more closely, and a single medication dose increase was made for isolated diastolic hypertension if it persisted after a week of SBP control. When pharmacotherapy was indicated, nifedipine XL was used first-line, followed by labetalol and enalapril. After 5 consecutive days of BP stability, monitoring frequency was reduced to weekly. BP ≥160/≥100 mm Hg or symptomatic hypotension triggered urgent clinical evaluation. Patients enrolled in the study continued to receive standard of care from their obstetrician as per ACOG guidelines. The primary outcome was postpartum hospital readmission within 6 weeks postpartum. Secondary outcomes were 6-week postpartum BP and frequency of medication titration. Feasibility was assessed through study retention and participant surveys. Where referenced, historical data were obtained from an observational cohort of 79 HDP patients (January-February 2020).

Sixty-two patients consented to enrollment (baseline characteristics in Table 1), of whom 23% had cHTN, 45% gHTN, and 31% preeclampsia (Table 2). Half of patients with cHTN and preeclampsia were discharged on antihypertensives, while 10% of those with gHTN were. Outcomes (Table 2) are reported among the 42 participants who completed the intervention. Seven percent of study patients were readmitted for hypertension, all within the first week postdischarge (as compared with 10% among the historical cohort). Ninety-five percent of study participants achieved an SBP of <130 mm Hg by the 6-week postpartum visit. Among the 41 patients in the historical cohort who had a BP ascertained at 6 weeks postpartum, 78% had a SBP <130 mm Hg. One-third of participants were initiated on antihypertensives after hospital discharge, and 62% received outpatient medication titrations through the study algorithm. The majority of titrations occurred in the first week postpartum for patients with gHTN, while patients with cHTN and preeclampsia required titration through the first 3 postpartum weeks. At 6 weeks postpartum, 31% remained on antihypertensives. There were no adverse hypotensive events. Medication side effects (flushing) were observed among 5/28 (18%) of patients on nifedipine, causing 4 to switch to either labetalol or enalapril. To achieve control, 50% of patients with cHTN and 23% with preeclampsia required more than a single agent, with 1 patient with preeclampsia requiring 3 simultaneous medications. At the study’s conclusion, only 1 participant (with cHTN) required 2 medications.

**Table 1:** Baseline Characteristics of Study Participants (N = 62)

Baseline demographics	
Maternal age, y	34 (32-37)
Self-reported ethnicity	
Hispanic or Latino	9 (14.5)
Not Hispanic or Latino	51 (82.3)
Not reported	2 (3.2)
Self-reported race	
White	43 (69.4)
Black/African American	6 (9.7)
Asian	4 (6.5)
Not reported	9 (14.5)
Private insurance	63 (85.5)
Language	
English	56 (90.0)
Spanish	6 (10.0)
Medical history	
Prepregnancy BMI, kg/m^2^	26.7 (22.3, 32.6)
Prepregnancy medical history	
Diabetes	3 (5.0)
Prior hypertensive disorder of pregnancy	8 (13.0)
Prior preterm delivery	3 (5.0)
Prior small for gestational age infant	3 (5.0)
Pregnancy information	
Parity	1 (0, 1)
Nulliparous	26 (42.0)
Primiparous	24 (39.0)
Multiparous	12 (19.0)
Mode of delivery	
Vaginal	39 (63.0)
Cesarean	23 (37.0)
Induction of labor	42 (65.0)
Weight gained during pregnancy, kg	12.45 (8.7, 16.0)
APGAR	
1 min	8 (7, 9)
5 min	9 (8, 9)
NICU admission	12 (18.0)
Medication history	
IV Antihypertensives during admission	10 (15.0)
Oral medication during admission	18 (28.0)
With additional IV treatment	9 (14.0)
Without additional IV treatment	9 (14.0)
Discharged on antihypertensive	19 (30.6)
NSAIDs prescribed at discharge	60 (96.8)

Values are mean (IQR) or n (%).

cHTN = chronic hypertension inclusive of patients who developed superimposed preeclampsia; gHTN = gestational hypertension.

**Table 2:** Baseline Characteristics and Outcomes of Participants Who Completed the Intervention

	Chronic Hypertension (n = 14)	Gestational Hypertension (n = 29)	Preeclampsia (n = 19)	Overall (n = 62)
Baseline characteristics				
Maternal age, y	34 (32, 36)	34 (30, 35)	36 (32, 40)	34 (32, 37)
Gestational age at delivery	37 (36, 39)	38 (37, 40)	37 (36, 38)	38 (37, 39)
Prepregnancy antihypertensive treatment	3 (21.4)	0 (0)	0 (0)	3 (4.8)
Prenatal antihypertensive treatment	4 (28.6)	0 (0)	0 (0)	4 (6.5)
Maximum inpatient systolic blood pressure (SBP), mm Hg	151 (126, 193)	140 (121, 170)	156 (118, 186)	147 (118, 193)
SBP at discharge	132 (104, 142)	120 (102, 144)	124 (100, 150)	124 (100, 150)
Discharged on antihypertensives	7 (50.0)	3 (10.3)	9 (47.4)	19 (30.6)

	Chronic Hypertension (n = 12)	Gestational Hypertension (n = 17)	Preeclampsia (n = 13)	Overall (n = 42)
Study outcomes				
Hospital readmission for HTN				
At first study call	0 (0)	0 (0)	1 (8.0)	1 (2.0)
Week 1	2 (17.0)	0 (0)	0 (0)	2 (5.0)
Antihypertensive initiation during study	5 (42.0)	5 (29.0)	4 (31.0)	14 (33.0)
Antihypertensive titration during study	10 (83.0)	7 (41.0)	9 (70.0)	26 (62.0)
Average medication changes per week among those requiring titration (# titrations/patient)				
Week 1	1.2	1.4	1.5	1.4
Week 2	0.8	0.4	1.3	0.9
Week 3	1.1	0.3	1.3	1.0
Week 4	0.2	0.3	0.1	0.1
Week 5	0.3	0.3	0.2	0.3
Week 6	0.2	0	0.4	0.3
Patients on antihypertensives at study completion	8 (67.0)	2 (12.0)	3 (23.0)	13 (31.0)
SBP <130 mm Hg at study completion	11 (92.0)	16 (94.0)	13 (100.0)	40 (95.0)
Primary care doctor seen within first postpartum year	9 (75.0)	12 (75.0)	11 (85.0)	32 (76.0)
Attended 6-wk postpartum visit with obstetrician	12 (100.0)	16 (100.0)	13 (100.0)	42 (100.0)

Values are median (IQR) or n (%).

Study retention was 82% at week 1, 77% at week 2, 69% at week 3, and 68% at week 6. Thirty-nine participants provided feedback after study completion, of whom 87% would recommend the program and 82% felt that daily monitoring improved postpartum care. However, 15% reported that daily monitoring increased stress, and 8% reported that daily phone calls were inconvenient. Of the 18 patients who were eligible but declined study participation, 61% cited a lack of time, and 17% cited concerns regarding home BP monitoring.

In this pilot study, utilization of an algorithm targeting a SBP <130 mm Hg at 6 weeks postpartum resulted in 95% achievement without adverse hypotensive events. In addition, monitoring frequency was designed to be significantly greater than currently recommended by ACOG and demonstrated a need for high-frequency clinical intervention in weeks 1 to 3 after hospital discharge.^1^ The BP target was chosen to align with ACC/AHA recommendations for nonpregnant patients, especially in light of mounting evidence that HDP increases later-life cardiovascular disease risk, largely mediated through hypertension.^2^^,^^3^ Indeed, a significant proportion (31%) of participants required antihypertensives at 6 weeks to achieve normotension as defined by the ACC/AHA.

Among patients who completed the intervention, hospital readmission for severe hypertension occurred in 7%, all within the first week. A third required medication initiation, and 62% received further titration through the study, most frequently in the first postpartum week among those with gHTN and in the first 3 postpartum weeks among those with cHTN and preeclampsia. These data are important to consider for future studies in order to design interventions that will promote patient engagement with the healthcare system (virtually or in person) on the clinically highest-risk days after hospital discharge.

Finally, while ACOG recommends that longitudinal BP management transition to a primary care provider (PCP) after 6 weeks postpartum, only 71% of HDP patients attended their 6-week postpartum obstetrician visit, and 46% saw a PCP within the first postpartum year among our historical cohort. In contrast, 100% of study participants who completed the study intervention attended their 6-week postpartum visit, and 76% saw their PCP within the first postpartum year, highlighting continued healthcare engagement that persisted beyond the study intervention. Given that patients with HDP are at high risk for future cardiovascular disease, strategies to promote healthcare engagement are important to improving primary prevention in this population.

As a pilot study, the primary limitation was small sample size. Larger, randomized studies among diverse maternal cohorts are needed to determine the frequency of BP monitoring needed to maximize patient engagement and achieve postpartum BP control. Despite attempts to remove barriers for participation, including native language speakers for Spanish-speaking participants, remote monitoring strategy (without need for travel or time investment for in-person study visits), and widely accessible technology for monitoring (ie, phone calls), only 68% of patients completed the study, demonstrating the need for further investigations into strategies to optimize patient engagement among diverse cohorts.

BP monitoring and medication titration to a SBP of <130 mm Hg resulted in 95% of participants achieving optimal BP control 6 weeks postpartum. A high proportion of participants required medication initiation and titration, predominately during the first 3 postpartum weeks. Future studies should further investigate strategies to better engage diverse cohorts of postpartum individuals.
